# A dysbiotic gut microbiome suppresses antibody mediated-protection against *Vibrio cholerae*

**DOI:** 10.1016/j.isci.2021.103443

**Published:** 2021-11-14

**Authors:** John C. Macbeth, Rui Liu, Salma Alavi, Ansel Hsiao

**Affiliations:** 1 Department of Microbiology and Plant Pathology, University of California, Riverside, Riverside, CA 92521, USA; 2 Division of Biomedical Sciences, School of Medicine, University of California, Riverside, Riverside, CA 92521, USA; 3 Graduate Program in Genetics, Genomics, and Bioinformatics, University of California, Riverside, Riverside, CA 92521, USA

**Keywords:** Disease, Microbiology, Microbiome

## Abstract

Cholera is a severe diarrheal disease that places a significant burden on global health. Cholera’s high morbidity demands effective prophylactic strategies, but oral cholera vaccines exhibit variable efficacy in human populations. One contributor of variance in human populations is the gut microbiome, which in cholera-endemic areas is modulated by malnutrition, cholera, and non-cholera diarrhea. We conducted fecal transplants from healthy human donors and model communities of either human gut microbes that resemble healthy individuals or those of individuals recovering from diarrhea in various mouse models. We show microbiome-specific effects on host antibody responses against *Vibrio cholerae*, and that dysbiotic human gut microbiomes representative of cholera-endemic areas suppress the immune response against *V. cholerae* via CD4+ lymphocytes. Our findings suggest that gut microbiome composition at time of infection or vaccination may be pivotal for providing robust mucosal immunity, and suggest a target for improved prophylactic and therapeutic strategies for cholera.

## Highlights

• Microbial variation of US donors drives divergent antibody responses to *V. cholerae*
• A dysbiotic model microbiota suppresses immune responses to *V. cholerae*
• CD4+ cell depletion restores protection in mice with a dysbiotic microbiota

## Graphical abstract

**Figure:**
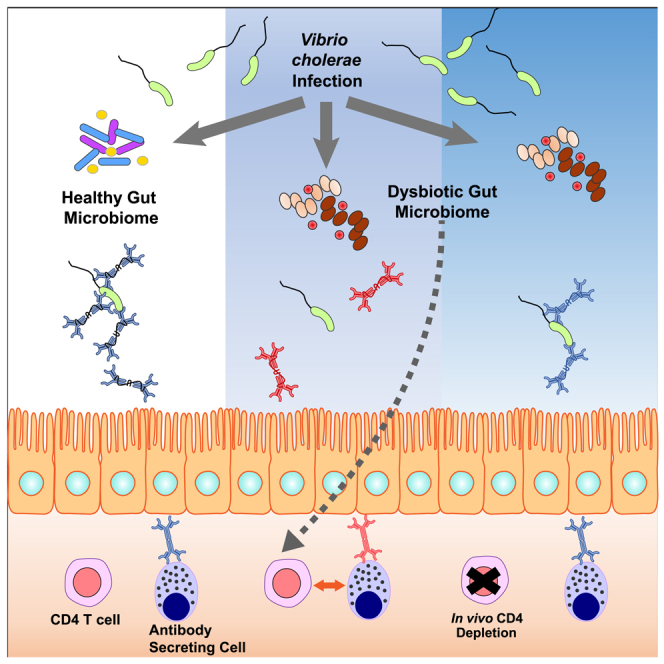

## Introduction

*Vibrio cholerae* is the etiologic agent of cholera, a severe diarrheal disease affecting approximately 3 million people annually, resulting in approximately 100,000 deaths (Ali et al., 2015). The bacterial mechanisms through which *V. cholerae* causes infection *in vivo* have been extensively studied. *V. cholerae* preferentially colonizes the small intestine, where it releases cholera toxin (CT), which causes profuse watery diarrhea and loss of electrolytes. While the advent of oral rehydration therapy has dramatically reduced mortality from cholera, recent major outbreaks are reminders of the pressing global public health need to improve cholera prevention strategies. Although cholera is thought of as a non-inflammatory disease, potentially because of the action of the MARTX toxin in suppressing host inflammatory responses during infection (Woida and Satchell, 2020), the relationship of host immunity to cholera is of critical importance to the control of the disease, both for the outcome of infection but also the outcome of prophylactic strategies such as vaccination. Though several oral cholera vaccines (OCVs) have been developed, they have demonstrated high variance in protective efficacy in field trials (Levine, 2010); however, OCVs have been shown to have protective efficacy of generally 80–90% in areas of good sanitation such as the United States and Europe, large field studies in cholera-endemic regions with less developed infrastructure such as Bangladesh and India have exhibited less overall efficacy of as little as 55% (Bishop and Camilli, 2011; Clemens et al., 1990a, 1990b; Harris, 2016; Levine et al., 1988; Richie et al., 2000). Cholera vaccine studies to optimize vaccine responses in endemic areas are ongoing, whether it be higher dosing of a live attenuated vaccine (Sow et al., 2017) or to understand the effects of single doses of killed oral cholera vaccines (Ali et al., 2021; Qadri et al., 2016, 2018). We hypothesized that one contributor to this high level of geographical variation in OCV efficacy, and potentially antibody responses to *V. cholerae* infection, may be variation in the microbial populations of the gut, the gut microbiome.

Several studies have shown that gut bacterial populations can change because of diet and geography (Subramanian et al., 2014; Yatsunenko et al., 2012), especially when comparing populations in the United States and Europe versus those in resource limited regions that have higher rates of enteric disease (Arumugam et al., 2011; Costea et al., 2018; Qin et al., 2010). After cholera diarrhea, the gut microbiome shifts to a taxonomically less diverse and dysbiotic state, largely composed of Streptococci, before transitioning to a conformation comparable to non-diarrheal individuals over the course of several weeks once the acute phase of the disease is over (Hsiao et al., 2014). This dysbiotic configuration has been observed in studies examining other enteric pathogens such as enterotoxigenic *E. coli* and rotavirus (David et al., 2015; Kieser et al., 2018) as well as other environmental insults such as malnutrition also commonly found in cholera-endemic areas (Subramanian et al., 2014). Recent work highlights the role of the gut microbiome in either conferring resistance or susceptibility to *V. cholerae* infection; key commensal microbes have been shown to modulate resistance to *V. cholerae* infection via degradation of bile salts, which are critical in the virulence activation pathway (Alavi et al., 2020). Although the presence of a normal murine microbiome has been implicated in antibody responses against viral vaccines (Oh et al., 2014), the effects of a human gut microbiome on host responses to *V. cholerae* or other enteropathogenic bacteria have not been well determined.

In this study, we sought to understand how variation in microbial communities affects immune responses upon infection with *V. cholerae.* We hypothesized that microbial dysbiosis from recurring environmental insults in cholera-endemic areas represents a recurring window of vulnerability to insufficient commensal-modulated immune responses to *V. cholerae*, and more broadly that interpersonal variation in microbiome structure can lead to individual-specific responses. We initially conducted fecal transplants from a small subset of human donors into germ-free mice in order to characterize immune correlates of protection when colonized with complete human fecal microbiomes. Based on these results, we moved beyond our fecal transplant observations to defined human model microbial communities in antibiotic-cleared mice. This allowed us to better understand the role of how interpersonal human microbiome variation at time of infection affects antibody responses. We show here that individual human gut microbiomes drive differential antibody responses to both wild-type and vaccine strains of *V. cholerae*, and that these immune responses are dampened by the presence of dysbiotic gut bacterial populations in a CD4+-cell-dependent manner. These findings suggest that gut bacterial composition at time of infection may impact adaptive immune responses to *V. cholerae*, and suggests that oral cholera vaccine design and distribution may need to take into account gut microbiome structure for optimal efficacy.

## Results

### Human microbiomes drive variable immune responses to *V. cholerae* in an adult germ-free mouse model of infection

Preclinical studies in animal models are essential to elucidate the mechanisms underlying interactions of host immunity, pathogens, and commensal microbes during infection. Several animal models have been developed for studying the behavior of *V. cholerae in vivo,* the most widely used being the infant mouse cholera model (Klose, 2000). However, although the suckling animals can be used to study *Vibrio* colonization and virulence, they are poorly suited for immunological studies, as the infant mouse does not have a fully developed adaptive immune system, a limitation shared by the recently developed infant rabbit model of cholera (Hubbard et al., 2018; Ritchie et al., 2010). Although adult conventionally-reared mice have been used to explore immune responses to infection (Nygren et al., 2008, 2009), the murine gut microbiome differs dramatically from human gut commensal communities, and is highly refractory to the addition of human-associated bacterial species (Seedorf et al., 2014). Adult germfree (GF) mice can be used to finely control microbial content, but exhibit reduced adaptive immunity in their axenic state. However, transient colonization with even a single bacterial species has been shown to restore immunoglobulin production to conventional levels (Hapfelmeier et al., 2010).

To evaluate the role of variation in human gut microbiomes in immune responses to *V. cholerae* infection, we used several experimental paradigms involving complete and defined model human gut microbiomes in adult mice. First, we transplanted several complete human fecal gut microbiomes into C57/BL6Tac germfree (GF) mice via intragastric gavage. These fecal samples were part of a previously-established biospecimen repository consisting of fecal samples taken from a healthy adult cohort in the United States (Alavi et al., 2020). Individuals were 18–45 years of age, and at time of collection had not suffered recent diarrhea or antibiotic usage, and did not report any ongoing inflammatory conditions of the gastrointestinal tract.

**Figure 1:**
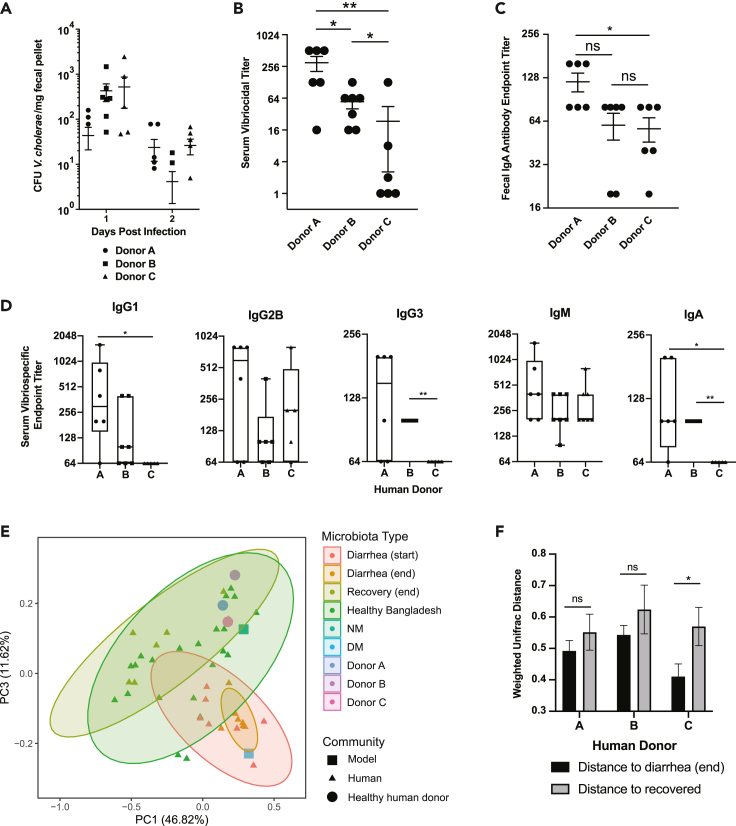
*V. cholerae-*specific antibody levels in fecal and serum samples (A) *V. cholerae* colonization in mice containing fecal microbiota of indicated human donors. (B) Serum vibriocidal titer in mice by donor community colonization after *V. cholerae* infection at 4 weeks post infection. (C) Fecal vibriospecific IgA endpoint titer in germ-free mice for human donors at 4 weeks post infection. (D) Serum vibriospecific endpoint titers for various types of antibody across the donors 4 weeks post infection. (E) Meta-analysis of existing 16S ribosomal gene sequencing datasets to compare Donors A, B, and C as well as defined communities (NM & DM) to an adult cholera cohort from Bangladesh and a cohort of healthy adults based on weighted UniFrac distance, % variance explained by axis shown in parentheses. Ellipses represent 95% confidence intervals. (F) Weighted Unifrac distances of US human donor communities versus Bangladeshi communities at diarrhea end or state of recovery ∗, p < 0.05, ∗∗, p < 0.01, Mann-Whitney *U* test. Error bars represent mean ± SEM. (1F) ∗, p < 0.05, Student's t-test. See also Figure S1.

After two weeks to allow for the establishment of human microbial colonization and restoration of adaptive immune activity, animals receiving these complete human fecal microbial communities were equivalently colonized (Figure S1A) prior to inoculation with ∼5 × 10^9^ CFU of *V. cholerae* C6706 El Tor. *Vibrio* shedding following infection was low and consistent across human microbiome contexts (Figure 1A), perhaps because of the high density of pre-established commensals relative to *V. cholerae* inoculum and the lack of virulence-associated clearance mechanisms such as diarrhea that is a characteristic of *V. cholerae* colonization in adult animals (Freter, 1956; Olivier et al., 2007).

To evaluate the efficacy of anti-*V. cholerae* antibody responses, we examined levels of *Vibrio*-specific immunoglobulin responses in both serum and fecal samples from these animals. First, we used a serum vibriocidal assay, which is considered to be the best clinical correlate of protection for cholera (Haney et al., 2017; Harris, 2018; Qadri et al., 2005; Sow et al., 2017). The serum vibriocidal titer (SVT) from this assay is the reciprocal of the highest dilution of serum at which killing of *V. cholerae* is observed with the addition of exogenous complement. In humans, a clinically successful seroconversion as a result of vaccination is defined as a more than four-fold rise in serum vibriocidal titers compared to baseline pre-immune titer over two weeks, although there is no defined titer at which protection can be considered to be definitively achieved (Kanungo et al., 2015). Interestingly, after 4 weeks post infection, the serum vibriocidal titer data varied up to 12-fold in animals hosting gut microbiomes from different human donors, ranging from a mean titer of 300 in Donor A to 23.5 in Donor C. This suggested that gut microbial composition was a strong and personalized driver of immune responses to the introduction of *V. cholerae* (Figure 1B). Measurement of vibrio-specific serum antibodies using a whole-cell ELISA assay yielded statistically significant differences by donor both in IgA, IgG1, and IgG3 isotypes (Figure 1D).

Although the serum vibriocidal titer is an important correlate of immunity after infection or vaccination, actual protection to subsequent challenge is mediated by secreted immunoglobulin at the gut mucosa (Strugnell and Wijburg, 2010). During the course of infection, class-switching to IgA and the secretion of antigen-specific secretory IgA (S-IgA) serves as the main means of protection by binding to *V. cholerae* and preventing pathogen access to epithelium, and neutralizing cholera toxin (Apter et al., 1993). Recent studies have also indicated that anti-O-specific polysaccharide antibodies in sera from humans surviving cholera can agglutinate *Vibrio* and prevent motility (Charles et al., 2020), and that expression of a monoclonal human anti-LPS IgA1 in mice can provide passive protection to infants from milk (Baranova et al., 2020). For an up-to-date article of cholera immunity, please read Holmgren J, Trop. Med. Infect. Dis., 2021 (Holmgren, 2021). A recent study highlights the capacity of a monoclonal IgA antibody to inhibit *V. cholerae* motility, preventing access to the intestinal epithelium (Levinson et al., 2015). The bulk of IgA in the body is secretory IgA (S-IgA) secreted in gram quantities per day onto the mucosa (Macpherson et al., 2012). We observed differences in antibody titers of *Vibrio*-specific IgA across weight-normalized fecal suspensions from mice colonized with different human donors, matching the pattern seen in serum vibriocidal responses; mice colonized with Donor A microbes showed the highest fecal IgA responses to *V. cholerae*, and Donor C the lowest (Figure 1C).

### A comparative analysis of US donors with Bangladeshi cholera cohorts yields insights into overall gut microbiome structures

We performed a meta-analysis using Principal Coordinates Analysis (PCoA) of existing 16S ribosomal RNA gene sequencing datasets to compare the microbial community structure of these complex human fecal microbiomes to an adult cholera cohort from Bangladesh (Hsiao et al., 2014) and a cohort of healthy adults from Bangladesh (Subramanian et al., 2014) (Figure 1E). In accordance with previous studies (Hsiao et al., 2014), the early- (“diarrhea start”) and immediately post-diarrhea microbiome (“diarrhea end”) was distinct from the state in the same individuals after 3 months of convalescence from diarrhea (“recovery”). Recovery samples in turn resembled a broader cohort of individuals that were healthy at time of sampling (“Healthy Bangladesh”). Although the US Donors (A, B, and C) overlapped in microbiome structure with healthy Bangladesh adults and with recovered diarrhea patients, the diversity among the ostensibly continuously healthy Bangladesh cohort was extremely high, and in some cases overlapped with dysbiotic post-diarrhea microbiomes (Figure 1E). This suggests that if gut microbiome dysbiosis as a function of diarrhea or malnutrition affects host immune responses to subsequent *V. cholerae* infection or vaccination, that the population impact in cholera endemic areas may be even more significant than the variance observed in US microbiomes. Interestingly, the microbiome of Donor C, which yielded the weakest SVT when transplanted into GF mice, was the only complex US donor community to be more similar to the dysbiotic post-diarrhea state in Bangladesh (“diarrhea end”) than the same patients 3 months after recovery from acute diarrhea (“recovery”) (Figure 1F) using an abundance-weighted metric, weighted UniFrac distance.

### Colonization of model communities of normal or dysbiotic gut microbiota results in differential immune response outcomes in mice

**Figure 2:**
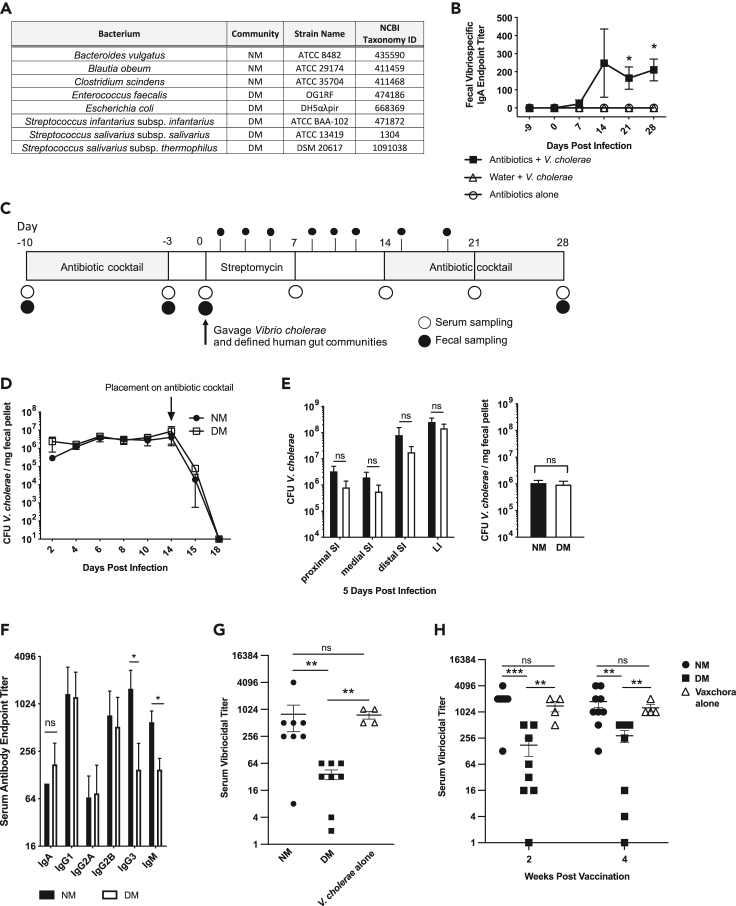
Model community colonization and antibody responses in antibiotic treated adult mouse model (A) Composition of defined human communities. (B) Antibiotic treatment is required to elicit vibrio-specific antibody responses. (C) Schematic of antibiotic treatment and bacterial introduction in SPF CD-1 mice. (D) *V. cholerae* colonization levels after co-gavage of NM and DM communities; antibiotic cocktail added at 14 days post infection. (E) *V. cholerae* colonization loads in proximal, medial, distal small intestine, large intestine, and fecal pellet at 5 days post infection. (F) Serum antibody profiles against whole cell *V. cholerae* 4-weeks post-infection. (G) Serum vibriocidal titer 4-weeks post-introduction of *V. cholerae* and indicated defined communities and *V. cholerae* alone in antibiotic-treated mice. (H) Serum vibriocidal titer 2 and-4-weeks post-vaccination with CVD 103-HgR-SmR in the presence of indicated defined communities and the vaccine strain alone in antibiotic-treated mice. NM: normal model microbiome, DM: dysbiotic model microbiome. SI: small intestine, LI: large intestine. ns, p > 0.05, ∗, p < 0.05, ∗∗, p < 0.01, ∗∗∗p < 0.001, Mann-Whitney *U* test. Error bars represent mean ± SEM. *n =* 4–8 mice per group for all experiments. See also Figures S1 and S2.

To expand upon our gnotobiotic studies we constructed several defined, simplified, model human gut microbiomes (Figure 2A) as shown in our previous studies (Alavi et al., 2020). One model community, “NM”, was characteristic of healthy human microbiomes found in the United States and Bangladesh, and contained *B. obeum* and a commonly found *Bacteroides, Bacteroides vulgatus*, and *Clostridium scindens*. As a comparison beyond “healthy” individuals, we constructed a second defined community (“DM”) that was representative of microbiomes suffering from dysbiosis found in cholera endemic areas (Alavi et al., 2020). A comparison against complex human microbiomes confirmed that the NM community was representative of healthy Bangladesh gut microbiomes, and the DM community was similar to the dysbiotic state found at the end of watery diarrhea (Figure 1E). Diarrhea of multiple etiologies, along with severe malnutrition, a common comorbidity of tropical diarrheas, drive the human gut microbiome to a characteristic low-diversity state dominated by bacteria such as Streptococci, Enterococci, and Proteobacteria (Alavi et al., 2020; David et al., 2015; Hsiao et al., 2014; Kieser et al., 2018; Subramanian et al., 2014). Although this state is able to recover over the course of weeks following the end of diarrhea or the application of therapeutic dietary interventions (David et al., 2015; Di Luccia et al., 2020; Hsiao et al., 2014), we hypothesized that this dysbiotic state represents a window where microbial community structure may lead to poor responses to *V. cholerae* antigen.

The logistical limitations of germfree mice limit the number and type of experimental human microbiomes and conditions to be tested. To address this in an immune-competent experimental system, we used adult conventionally reared CD-1 animals that had their native microflora cleared using treatment with antibiotics, as mouse-adapted microbes rapidly out-compete non-murine communities (Seedorf et al., 2014). Animals were given an antibiotic cocktail in drinking water for 1 week (See STAR Methods) and then switched to streptomycin treatment alone 3 days before the gavage. The mice were then gavaged with *V. cholerae* C6706 O1 El Tor, which is resistant to streptomycin as well as the respective NM or DM communities. As measured by 16S qPCR, total bacterial load was consistent between NM and DM and persisted at least until 48 h post gavage (Figure S1B). We observed that antibiotic treatment was in fact critical to observe robust antibody responses against infection by *V. cholerae* (Figure 2B).

Because even very transient presence of *V. cholerae* was able to induce strong antibody responses in a microbiome-dependent manner in GF mice, and to prevent any sustained differences in *V. cholerae* colonization, as well as the resurgence of murine commensals, we placed these animals back on an antibiotic cocktail after 2 weeks post introduction of *V. cholerae* (see Figure 2C for experimental layout). The extended presence of streptomycin and restoration of antibiotic cocktail in this experimental system prevented major effects of the host microbiomes on *V. cholerae* that might be expected from previous studies of the effect of human commensals on colonization resistance (Alavi et al., 2020), thus standardizing the amount of *V. cholerae* able to interact with host immunity across groups. In order to confirm that the load of *V. cholerae* does not contribute to subsequent immune outcomes, we measured CFU *V. cholerae* per mg fecal pellet including after placement on an antibiotic cocktail at 14 days post infection (Figure 2D). We observed no variation in *V. cholerae* levels in the mice that were given the NM or DM communities at time of infection. In addition, to evaluate whether *V. cholerae* colonization load may affect gut mucosal antibody responses, we examined proximal, medial, and distal small intestine as well as large intestine and fecal pellet *V. cholerae* colonization levels 5 days post infection while the mice were maintained on streptomycin and before replacement on the antibiotic cocktail and found no statistically significant differences in pathogen load in this system (Figures 2D and 2E). Taken together, these data suggest that any differences in host immune responses by the presence of model human microbes during infection will not be because of accessibility of antigen in a colonization-dependent manner.

At 4 weeks post introduction of *V. cholerae*, serum and fecal samples were collected from antibiotic-cleared mice containing NM and DM human microbiomes. Serum vibrio-specific ELISA showed that levels of IgG3 and IgM, strong complement fixing antibodies, were decreased in the DM group as compared to the NM group (Figure 2F). In addition, vibrio-specific antibody levels were examined at 0 days post infection and 2 weeks post infection but showed no significant differences as a function of microbiome at time of introduction of *V. cholerae* (Figure S2). We observed that serum from animals bearing the (DM) microbiome at time of infection exhibited a statistically significant reduction in serum vibriocidal activity compared to that from animals infected in the presence of the (NM) microbiome as well as *V. cholerae* alone (Figure 2G), suggesting that the presence of members of the dysbiotic community at time of infection may hinder the development of a robust serum antibody response.

### Fecal Ig from *V. cholerae* infected mice bearing the DM community are less protective in an infant passive protection mouse model

**Figure 3:**
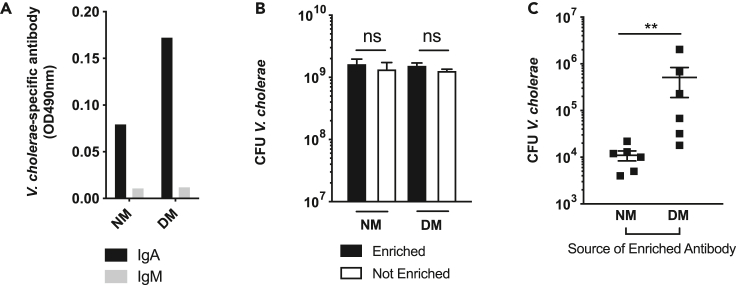
Enriched fecal antibody of infected NM, but not DM animals, can passively protect suckling animals from *V. cholerae* infection (A) Isotype distribution of pooled, enriched fecal antibodies from NM and DM mice. (B) Survival of *V. cholerae* incubated with either enriched or not enriched fecal antibody preparations for 6 h. (C) Colonization of suckling CD-1 mice by *V. cholerae* pre-incubated with enriched fecal antibody from infected mice bearing NM and DM microbiomes. Input was normalized so that *V. cholerae* used to colonize either NM and DM groups were incubated with equivalent amounts of IgA. ∗∗, p < 0.01, Mann-Whitney *U* test. Error bars represent mean ± SEM.

Although the vibriocidal assay represents an established correlate of protection in humans, we examined the ability of mucosal antibodies to affect *V. cholerae* infection, as secreted immunoglobulins are likely the direct mediators of protection following immunity because of natural infection or immunization. We therefore used a passive protection assay to determine the efficacy of fecal antibodies in protection against *V. cholerae*. Fecal antibodies generated by animals containing different model microbiomes were enriched from other fecal constituents using protein L purification (See STAR Methods). These antibody pools were predominantly IgA with low levels of IgM (Figure 3A). To examine whether enriched antibody preparations or fecal water had any intrinsic effects on *V. cholerae* growth, we conducted an *in vitro* growth inhibition assay. We observed no alteration in growth between enriched and unenriched fecal water for our respective communities, confirming the absence of inhibitory components in enriched antibody preparations (Figure 3B). Enriched Ig from both groups was combined with *V. cholerae* grown overnight and incubated for 1 h before being gavaged into 4-day old infant CD-1 mice. Suckling animals were used as conventionally-reared adult animals without antibiotics are highly resistant to *V. cholerae* colonization (Olivier et al., 2007, 2009). After 18 h of infection, the small intestines were homogenized and plated on selective medium. We observed that pre-treatment with antibody from animals bearing the dysbiotic microbiota led to colonization nearly 2-log greater than pre-infection treatment with antibody from animals with the NM microbiome (Figure 3C). Taken together, these findings suggested that oral infection in a DM microbiome context led to a significantly less effective anti-*Vibrio* antibody response.

### Live members of DM community exhibit dominant suppressive effects on antibody responses

**Figure 4:**
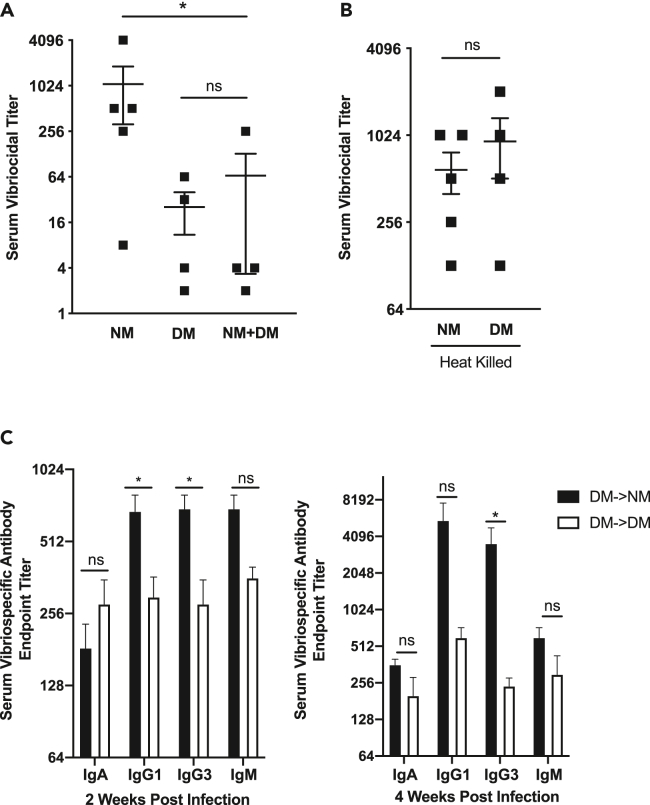
The effect of DM microbes is dominant on infection outcomes, and requires the presence of live bacteria during infection (A) Serum vibriocidal titers 4-weeks post-infection in CD-1 mice infected with *V. cholerae* and bearing indicated human model microbiomes. (B) Vibriocidal titers of mice gavaged with indicated heat-killed communities at time of infection with live *V. cholerae*. (C) Profiles of anti-whole cell *V. cholerae* serum antibody 2 weeks (left) and 4 weeks (right) post-infection in mice that were pre-colonized for 4 days with DM communities and subsequently given either NM or DM at time of infection. ∗, p < 0.05, Mann-Whitney *U* test. ns, p > 0.05, Mann-Whitney *U* test. Error bars represent mean ± SEM.

To determine whether the NM or DM anti-*Vibrio* antibody phenotype would be dominant when the bacterial communities are combined, we infected mice with *V. cholerae* in the presence of either NM, DM, or NM + DM microbiomes. At 4 weeks post infection, the NM + DM group showed a low serum vibriocidal titer comparable with the DM group, while the NM group had significantly higher titer levels than NM + DM (Figure 4A). These data suggested that the dysbiotic microbiome may have a role in suppressing the host antibody response against *V. cholerae.* Due to the reduced vibriocidal titer levels observed in the NM + DM group, we wanted to determine whether or not live members of the susceptible community were required to mediate this effect. Accordingly, we heat inactivated all the members of the respective communities and again infected mice with live *V. cholerae.* We observed that the serum vibriocidal titer increased in the heat-killed DM group were to similar levels with the NM group (Figure 4B), suggesting that live members of the dysbiotic community are necessary at time of infection to mediate suppression of anti-*Vibrio* antibody protection.

Although the DM microbiome exhibited a dominant reduced anti-*Vibrio* antibody phenotype in the NM + DM group, we wanted to determine whether the NM group could potentially rescue the DM phenotype under different circumstances. To study this, we initially introduced the DM community into antibiotic-treated animals 4 days prior to infection with *V. cholerae*. To model a targeted modification of the gut microbiome shortly after infection or immunization with OCVs, we either co-gavaged the NM with *V. cholerae* + NM or *V. cholerae* + DM. At 2- and 4-weeks post infection, we observed that serum vibrio-specific Ig was significantly increased in the DM- > Vc + NM group as compared to the DM- > Vc + DM group, suggesting that the presence of NM microbes was able to partially rescue the DM phenotype using specific treatment conditions (Figure 4C).

### Depletion of CD4+ cells restores *Vibrio-*specific immune response in mice colonized with DM defined community

**Figure 5:**
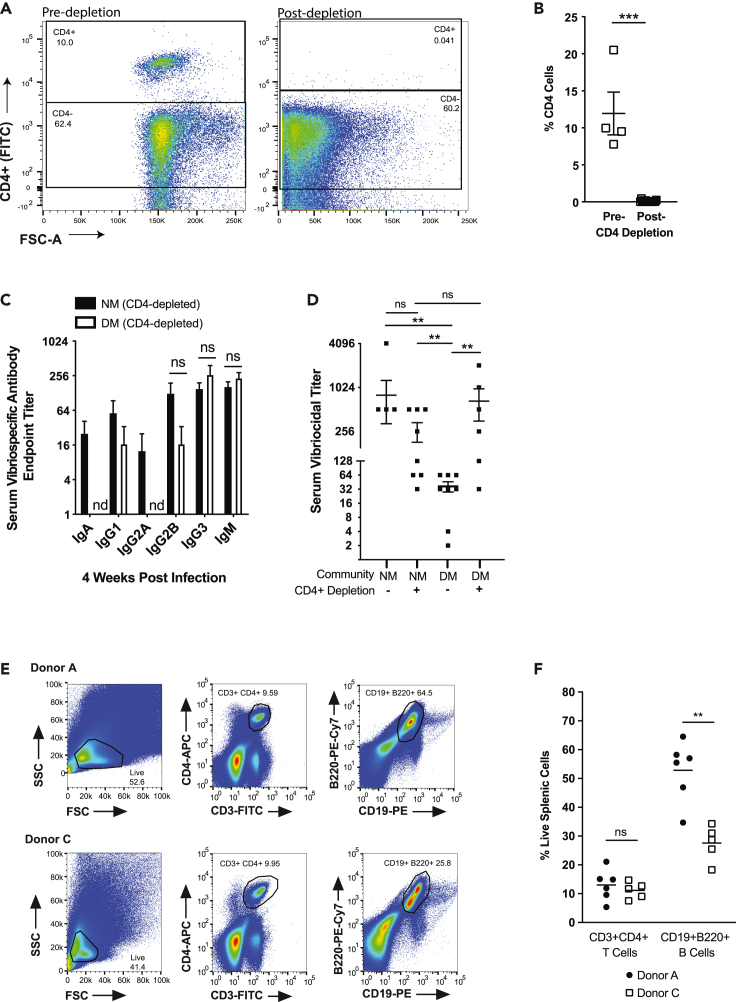
DM community effects are mediated *in vivo* by CD4^+^ cell populations (A and B) % of CD4 cells pre and 7-days post depletion in blood. (C) Serum antibody levels against *V. cholerae* 4-weeks post-infection in the presence of indicated human model microbiomes in CD4+ depleted mice. (D) Comparison of vibriocidal titer levels in NM and DM groups 4-weeks after infection with or without CD4^+^ cell depletion. (E and F) Analysis of splenic T cells and B cells in germ-free mice bearing human donor microbiomes. ∗, p < 0.05, ∗∗, p < 0.01, ∗∗∗, p < 0.001, ns, p > 0.05, Mann-Whitney *U* test, nd, not detected. Error bars represent mean ± SEM.

In general, immune responses to *V. cholerae*, whether in the context of infection or immunization, have yet to be fully elucidated. Initial OCV responses appear to be driven by TLR-2-dependent interactions that can cause CD4^+^ proliferation, and it has been shown in natural *V. cholerae* infection that CD4^+^ T cells are also instrumental in stimulating long-term memory B cell responses (Bhuiyan et al., 2009; Kuchta et al., 2011; Sirskyj et al., 2016; Weil et al., 2009). Even though the overall levels of T lymphocytes remained constant during colonization with different microbiomes, B-cell expansion depends on the action of numerous types of cells such as antigen-presenting dendritic cells, M Cells as well as CD4+ cells, including T_FH_ cells and T_Reg_ cells (Cerutti and Rescigno, 2008; Perez-Lopez et al., 2016). To determine if CD4+ cells were responsible for mediating immune system effects of different microbiomes, we used antibiotic-depleted mice bearing NM and DM model microbiomes under CD4+ cell depletion. We were able to ablate CD4+ cell populations through intraperitoneal injection with anti-CD4 monoclonal antibodies every 4 days during antibiotic treatment. After verifying depletion of CD4^+^ cells by flow cytometry analysis of whole blood (Figures 5A and 5B), animals were gavaged with live defined microbial communities and *V. cholerae* as previously described. Levels of serum anti-*V. cholerae* IgA were severely reduced in both groups compared to non-depleted animals. Similarly, serum anti-*V. cholerae* IgG3 and IgM were decreased in the NM group compared to non-depleted animals (Figures 2A and 5C). Depletion of CD4+ cells yielded no statistically significant differences in levels of serum IgG and IgM, but strikingly, the vibriocidal titer of the DM group increased to levels comparable to the NM group after CD4^+^ cell depletion (Figure 5D). This level was also comparable to that observed in NM group without depletion, suggesting that CD4^+^ cells are not required for the development of serum vibriocidal responses, and interactions between these host cell populations and members of the dysbiotic gut microbiome leads to suppression of subsequent development of specific antibody responses.

### Interpersonal microbiome variation results in variable splenic B cell populations

We next extended these studies to GF mice colonized with complex human donor microbiomes and challenged with *V. cholerae.* We focused our studies on mice with donor A and C microbiomes, as these communities were associated with the highest and lowest vibriocidal antibody titers upon *V. cholerae* introduction. In animals with A and C donor microbes, levels of splenic T-lymphocytes (CD3+ CD4+) were not statistically significant (Figures 5E and 5F). However, we observed higher levels of CD19+ B220+ B-cells in spleens of donor A mice compared to Donor C animals (Figures 5E and 5F). This is consistent with the higher levels of fecal and serum antibodies generated by donor A mice in contrast to donor C mice. These data are strong indicators of the influence of microbial communities on impacting immune responses to *V. cholerae*.

### Suppressed immune response in DM mice given modified CVD 103-HgR

To broaden the applicability of our previous observations detailing microbiome compositional changes affecting immune outcomes to *V. cholerae* infection in mice, we utilized the strain used in the FDA-approved live-attenuated vaccine Vaxchora. CVD103-HgR is an O1 Inaba strain containing a 94% deletion of the cholera toxin enzymatic subunit gene *ctxA* and shows high immunogenicity in US populations (Chen et al., 2016). We isolated an isolate of CVD103-HgR demonstrating spontaneous resistance to streptomycin (CVD 103-HgR-SmR) for inoculation into our antibiotic treated adult mouse model. Using CVD 103-HgR-SmR, we observed similar immune responses in the NM and DM groups as compared to infection with wild-type C6706, albeit at 2 weeks post vaccination (Figure 2H). The DM group given the vaccine strain showed a 7-11-fold decrease in SVT as compared to mice given the NM communities and a 4-8-fold decrease as compared to mice only given CVD 103-HgR-SmR. More studies will need to be carried out to further the observations in these results; however, these data describe the impact transient colonization of the gut microbiome impacts downstream vaccine-specific antibody responses.

## Discussion

In humans, the gut microbiome enters a DM-like state transiently after infectious diarrhea or severe malnutrition, because of repeated infection by multiple pathogens, ranging from cholera to pathogenic *Escherichia coli* and rotavirus, a state that is likely to be much more frequently attained in cholera-endemic areas (David et al., 2015; Hsiao et al., 2014; Subramanian et al., 2014). Malnutrition, another common public health concern often co-occurring with recurrent infectious diarrhea, induces a DM-like state for much longer periods, and is refractory to therapeutic nutritional intervention (Smith et al., 2013). Previous work has demonstrated that this transient DM-like state represents a risk factor for *V. cholerae* colonization (Alavi et al., 2020). Our work suggests that this dysbiosis may also represent a risk factor for poor immune responses to *V. cholerae* beyond infection; the composition of the human gut microbiome at time of exposure to *V. cholerae* in antibiotic-cleared and GF animals can suppress resultant antibody-mediated immune responses.

These findings also have significant implications for the use of OCVs. Efficacy of vaccination against enteric pathogens has been shown to be highly variable on a geographical and per-study basis, including for rotavirus (Harris et al., 2017), *Salmonella* (Eloe-Fadrosh et al., 2013), polio (Huda et al., 2014) and cholera (Levine, 2010). One of the potential reasons for the variability may be due to interpersonal variations in gut microbiomes (Sack et al., 2008). Previous studies sought to identify the relative abundance of certain species that were either positively or negatively correlated with protection from infection (Midani et al., 2018), but few studies have examined how microbiome composition affects host immune responses to infection in experimental models. We observed in our studies that individuals who were healthy overall and whose microbiomes aligned well with the healthy Bangladeshi group, exhibited variable immune response outcomes in our germ-free mouse model. In order to move to a more experimentally tractable, reductionist animal model, we designed simple model communities that are representative of gut microbiomes present in healthy versus diarrhea endemic populations.

Our DM model community is similar to these dysbiotic microbiomes in humans both by overall community diversity and types of characteristic organisms; human microbiomes during fulminant diarrhea and early recovery from diarrhea can be dominated by 99% *Streptococcus* species by relative abundance (Hsiao et al., 2014). Live, as opposed to heat-killed, DM community organisms were able to suppress serum and fecal antibody responses to introduction of *V. cholerae*. One prior study shows that *Sutterella* species are capable of degrading the stabilizing peptide of s-IgA, leading to decreased levels of IgA (Moon et al., 2015). The mechanism in our studies is likely different, as our microbial populations are only transiently present, and overall antibody levels are comparable across different model microbiomes. Our results suggest that even brief differences in microbiome structure may have important consequences, for example in OCV effectiveness, where microbiome dysbiosis at time of immunization can jeopardize outcomes (Levine, 2010).

This suppressed host antibody response can be reversed in DM-colonized animals through subsequent microbiome modification by microbes that are more characteristic of the healthy human gut microbiota. Although the definition for what specific taxa constitutes a truly “healthy” microbiome is not settled, our NM model community is very broadly reflective of healthy human communities at higher taxonomy levels and by PCoA analysis. The introduction of the NM community at time of *Vibrio* infection of mice colonized by DM microbes was able to partially rescue the generation of robust anti-*Vibrio* serum Ig. This has significant translational implications as it suggests that a normal microbiota consortium may be used to improve OCV outcomes.

Our antibiotic treated adult mouse experimental system is a robust model to study gut microbiota interactions in the host. In contrast to previous studies (Butterton et al., 1996), we are able to transplant actual human microbes into an immune-competent animal system, shortening the loop from initial observations to potentially clinically-relevant conclusions. Results with simplified defined microbiomes, similarly to complex human fecal microbiomes in germfree mice, exhibited interpersonal/inter-community differences in driving anti-*Vibrio* immune responses. However, additional human fecal communities, including those from cholera endemic areas, will be necessary to more robustly probe temporal variations in interactions between the host and the broad range of microbiome structures seen in healthy humans induced by temporal and intrapersonal variation in individuals with complex microbiomes.

Unlike other enteric pathogens such as *Shigella* and *Salmonella*, which cause clinically apparent inflammation and disease after penetrating cells or the intestinal epithelium, *V. cholerae* is thought to cause a non-inflammatory, noninvasive infection. However, cholera is associated with inflammatory changes such as widening of intracellular spaces, apical junction abnormalities as well as an infiltration of neutrophils, mast cells, and macrophages into the affected area (Mathan et al., 1995; Pulimood et al., 2008). Although innate immune cells such as neutrophils were shown to be essential for containment of *V. cholerae,* protection is mainly derived from adaptive immunity (Queen and Satchell, 2012). To begin teasing apart the host mechanism behind our observed microbiome-dependent antibody response phenotypes, we examined the role of CD4^+^ T cells, which are important cellular regulators of B cell maturation into antigen specific IgA secreting plasma cells (Cerutti and Rescigno, 2008). Upon depletion of CD4^+^ cells, we observed decreased levels of serum IgA after infection in both NM and DM mice, potentially indicating decreased seroconversion (Figure 5C). However, serum vibriocidal titer in DM, CD4^+^-depleted animals increased to levels comparable to the NM mice (Figure 5D). These data show that CD4^+^ cells are integral in mediating microbiome-dependent changes in an infection-induced antibody response. These results are surprising as one would expect CD4^+^ T cell depletion to substantially reduce the vibriocidal titer but our data suggests that there are compensatory, non-CD4^+^ mediated mechanisms to aid in seroconversion. A recent clinical study evaluating the efficacy of the oral cholera vaccine Shanchol in human immunodeficiency virus (HIV)-infected individuals demonstrated that while vibriocidal titer was lower in HIV-infected individuals with depleted CD4^+^ T cell populations, there was still seroconversion in 65–74% of the subjects (Ivers et al., 2015). Although the study population was not completely depleted of CD4^+^ T cells, it demonstrates vibriocidal titers can be elicited even in a highly-CD4^+^ cell-depleted state, albeit to a lesser degree. Furthermore, although our analysis of splenic T cell populations yielded no differences as a function of complex donor microbiome colonization, our phenotype may depend on change in certain specific T cell types such as Regulatory T cells (T_REG)_ or Follicular Helper T cells (T_FH_); further experimentation will be required to define these specific T cell subtypes. Interestingly, there was an increase in splenic B cells in the mice given fecal transplants from Donor A as compared to Donor C, indicating that the gut microbial community in Donor A was associated with more robust immune responses including highly proliferative B cell populations.

We extended our observations from wild-type C6706 *V. cholerae* to a live-attenuated vaccine strain, Vaxchora. Because the native murine microbiome is refractory to *V. cholerae* colonization (Freter, 1956), we utilized an isolate of CVD 103-HgR that was spontaneously resistant to streptomycin. The DM and NM communities are associated with similar host SVT responses to the vaccine strain as with wild-type *V. cholerae* (Figure 2H). In addition, the NM community shares a similar SVT profile to the vaccine strain alone, suggesting that NM does not significantly boost response above that of the vaccine strain. Our studies in mice reflect a recent human clinical study that compared SVT data in age matched North America and Bangladesh adults that were voluntarily infected with *V. cholerae* O1 Inaba. Notably, anti-CtxB IgA and IgM responses were greater in the North American group compared to the Bangladeshi participants (Hossain et al., 2019). These findings support the notion that specific human gut microbial populations can result in varied humoral immune responses to *V. cholerae.* Antibody-mediated protection to natural infection is both anti-toxin and anti-bacterial cell (Weil et al., 2019), whereas vaccine-mediated immunity is predominantly against LPS (Svennerholm, 1975). Although much remains to be elucidated in relation to the effects of the gut microbiome on cholera vaccine responses, our data adds to the ever-increasing literature of the role of gut bacteria modulating mucosal vaccine immune responses.

To more fully understand the correlations between bacterial communities, *V. cholerae*, and host interactions, more work will need to be done to study the biochemical underpinnings of microbiome-host interaction as it impacts host immunity. The precise molecular interface between DM microbes and the immune system is yet to be defined; the inability of heat-killed DM communities to influence infection outcomes suggest that an active interaction with host tissue, or the production of active compounds *in vivo* are required for this. At the host level, although we investigated the role of CD4^+^ T cells in this phenotype, other immune cell types such as antigen-presenting cells may act as more direct intermediaries between host immunity and microbial composition. As mentioned previously, Helper T cells are integral in stimulating and guiding B cell responses, so it would be beneficial to further define CD4^+^ subsets involved such as follicular helper T cells or regulatory T cells as well as B-cell subtypes.

Taken together, our data advances how gut microbiome structure may alter the immune pathways resulting in a weakened humoral response. Ultimately, our studies on the influence of bacterial composition at time of introduction of *V. cholerae* to the gastrointestinal tract will help delineate the host contributors to infection response, as well as the immune response to introduced antigen such as with live attenuated OCVs. Variability in the gut microbiome may thus contribute to both individualized disease outcomes, and the high observed variability in oral cholera and other mucosal vaccines.

### Limitations of the study

Although our data further advances the understanding and impact of gut bacterial composition on immune outcomes to natural infection with *V. cholerae* or vaccination, it is important to acknowledge several limitations to our study's approach and animal modeling. Although we examined immune correlates of protection of cholera from US stool donors in germ-free mice, it would be an informative comparison to do a similar analysis with stool samples from populations where enteric disease is common. In addition, mouse models are an imperfect lens through which human disease and immune biology can be viewed. Some human-associated microbes do not successfully engraft into the mouse gut, and the distribution of these microbes vary from rodent to human, especially in complex fecal microbiomes. To gain more translational insights, further analyses and modulation of complex human fecal microbiomes in the context of OCV administration would ultimately be required.

## STAR★Methods

### Key resources table

**Table:** 

REAGENT or RESOURCE	SOURCE	IDENTIFIER
**Antibodies**		

InVivoMab anti-mouse CD4 antibody Clone GK1.5	BxCell	Cat#BE0003-1; RRID: AB_1107636
Rat anti-mouse CD16/32 Clone 2.4G2	BD Pharmingen	Cat#553142; RRID: AB_394657
APC anti-mouse CD4 Clone RM4-5	Invitrogen	Cat#17-0042-82; RRID: AB_469323
FITC anti-mouse CD4 Clone RM4-5	Invitrogen	Cat#11-0042-82; RRID: AB_464896
FITC rat anti-mouse CD3 Clone 17A2	BD Pharmingen	Cat#561798; RRID: AB_10898341
PE rat anti-mouse CD19 Clone 1D3	BD Pharmingen	Cat#557399; RRID: AB_396682
PE-Cy7 Anti-mouse B220 Clone RA3-6B2	Invitrogen	Cat#25-0452-82; RRID: AB_469627
Goat anti-mouse IgA-HRP	Southern Biotech	Cat#1040-05; RRID: AB_2714213
Goat anti-mouse IgG1-HRP	Southern Biotech	Cat#1071-05; RRID: AB_2794426
Goat anti-mouse IgG2A-HRP	Southern Biotech	Cat#1081-05; RRID: AB_2736843
Goat anti-mouse IgG2B-HRP	Southern Biotech	Cat#1091-05; RRID: AB_2736842
Goat anti-mouse IgG3-HRP	Southern Biotech	Cat#1101-05; RRID: AB_2794588
Goat anti-mouse IgM-HRP	Southern Biotech	Cat#1021-05; RRID: AB_2794240

**Bacterial and virus strains**		

*Vibrio cholerae* C6706 El Tor	Hsiao Lab stock	C6706
CVD 103-HgR-SmR	This paper	Vaxchora/CVD 103-HgR-SmR
*Escherichia coli*	Hsiao Lab stock	DH5α- λpir
*Streptococcus salivarius* subsp. *salivarius*	ATCC	ATCC 13419
*Blautia obeum*	ATCC	ATCC 29174
*Clostridium scindens*	ATCC	ATCC 35704
*Bacteroides vulgatus*	ATCC	ATCC 8482
*Streptococcus infantarius* subsp. *infantarius*	ATCC	ATCC BAA-102
*Streptococcus salivarius* subsp. *thermophilus*	DSMZ	DSM 20617
*Enterococcus faecalis*	Hsiao Lab Stock	OG1RF
*Escherichia coli*	Hsiao Lab Stock	BW30045

**Biological samples**		

Human volunteer donor fecal sample	Alavi, et al., (2020)	Donor A
Human volunteer donor fecal sample	Alavi, et al., (2020)	Donor B
Human volunteer donor fecal sample	Alavi, et al., (2020)	Donor C

**Chemicals, peptides, and recombinant proteins**		

Ampicillin sodium salt	Fisher Bioreagents	Cat#BP1760
Neomycin trisulfate salt hydrate	Sigma-Aldrich	Cat#N1876
Vancomycin hydrochloride	Alfa Aesar	Cat#J62790.06
Streptomycin sulfate	VWR Life Sciences	Cat#0382
Guinea pig complement serum	Sigma-Aldrich	Cat#234395
Critical commercial assays		
iQ SYBR Green Supermix	Biorad	Cat#170882
SuperScript IV First-Strand Synthesis System	Invitrogen	Cat#18091200
Protein L Purification Kit	ThermoScientific	Cat#88849

**Deposited data**		

Short-read sequencing data for meta-analysis	European Nucleotide Archive	See Table S1 for accession numbers
Experimental models: Organisms/strains		
Mouse: C57/BL6-Tac Inbred	UCR gnotobiotic facility	N/A
Mouse: CD1 IGS	Charles River Laboratories	N/A

**Oligonucleotides**		

16S F PCR Primer forward: 5’-CTCCTACGGGAGGCAGCAG-3’	IDT	N/A
16S R PCR Primer reverse: 5’-TTACCGCGGCTGCTGGCAC-3’	IDT	N/A

**Software and algorithms**		

QIIME	Caporaso et al. (2010)	http://qiime.org/
Graphpad Prism	Graphpad software (CA, USA)	N/A
FlowJo	BD Biosciences	N/A

**Other**		

Lab diet	Newco Distributors	Cat# 5K52

### Resource availability

#### Lead contact

Further information and requests for resources should be directed to and will be fulfilled by the lead contact, Ansel Hsiao (ansel.hsiao@ucr.edu).

#### Materials availability

Unique strains and reagents generated in this study are available from the lead contact with a completed Materials Transfer Agreement.

### Experimental model and subject details

#### Animal and human studies

Female CD-1 mice were purchased from Charles River Laboratories, and generally used at 5-9 weeks of age. 4-day old suckling CD-1 mice were purchased from Charles River Laboratories. Germfree C57/BL6Tac animals were bred and reared in the gnotobiotic facility at the University of California, Riverside. Male and female C57/Bl6Tac mice were used generally at 5-9 weeks of age. No differentiation was observed between sexes and animal data were pooled by sex where applicable. Animals in the study were treated and housed under specific-pathogen-free or germfree conditions. All animal protocols were approved by University of California, Riverside’s Institutional Animal Care and Use Committee. All human samples were part of a study approved by the University of California, Riverside’s Institutional Review Board.

#### Human study design and sample collection

Human stool samples from a cohort of healthy adult individuals were collected at the University of California, Riverside using an IRB-approved protocol. Inclusion criteria were: between 18-40 years old, ability to provide informed consent, and willing and able to provide a stool specimen. Exclusion criteria were: systemic antibiotic usage (oral, intramuscular, or intravenous) 2 weeks prior to stool collection, acute illness at time of enrollment, diarrhea or very loose stools within 2 weeks prior to collection, active uncontrolled GI disease such as Crohn’s disease, ulcerative colitis, gastritis, constipation, major surgery of the GI tract (excluding cholecystectomy and appendectomy). Fecal samples were stored at −80°C until further processing. Stocks of fecal slurries for subsequent experimentation were prepared by re-suspending samples at 1:3 weight/volume in sterile reduced PBS and adding sterile glycerol to a final concentration of 25% volume/volume.

### Method details

#### Germ-free and gnotobiotic mouse experiments

Germ-free C57/BL6Tac mice were bred and maintained in plastic gnotobiotic isolators at University of California, Riverside. Mice were fed an autoclaved, low-fat plant polysaccharide-rich mouse chow (Lab Diet 5K52) and were 6-13 weeks old at time of gavage. We used real-time PCR and universal 16S primers to normalize human fecal slurries so that each adult mouse received approximately 20 μg of microbial genomic DNA. Reactions comprised 2 μL of extracted DNA (200 ng/reaction) as template, 12.5 μL SYBRGreen Master Mix (BioRad), 10 μL PCR-grade water, and 0.25 μL of forward and reverse primers at 10 μM (forward: 5’-CTCCTACGGGAGGCAGCAG-3’, reverse: 5’-TTACCGCGG CTGCTGGCAC-3’). Cycle conditions were 95°C for 3 min, followed by 39 cycles (95°C for 10 sec, 55°C for 30 sec, 95°C for 10 sec, 65°C for 5 sec, 95°C for 5 sec). Mice were fasted for two hours prior to introduction of bacteria, and stomach pH was buffered by intra-gastric gavage of 100 μL 1 M NaHCO_3_, followed by gavage with 150 μL of fecal slurries. 2 weeks after human commensal colonization, each group was infected with ∼5 x 10^9^ CFU *V. cholerae* O1 El Tor C6706. Fecal samples were suspended in 500 μL of PBS and homogenized using a bead beater (BioSpec) at 1,400 RPM for 30 seconds. CFU enumeration of *V. cholerae* was done on LB agar containing 200 μg/mL streptomycin.

#### Bacterial strains and growth conditions

All human gut commensal strains used are listed in Figure 2A. Unless otherwise noted, human gut strains were propagated in LYHBHI liquid medium (BHI supplemented to 5 g/L yeast extract, 5 mg/L hemin, 1 mg/mL cellobiose, 1 mg/mL maltose and 0.5 mg/mL cysteine-HCl). Cultures were then grown in a Coy anaerobic chamber (atmosphere 5% H_2_, 20% CO_2_, balance N_2_) or aerobically at 37°C. All *V. cholerae* strains were derived from the C6706 El Tor pandemic isolate and propagated in LB media with appropriate antibiotics at 37°C. Vaxchora (CVD 103-HgR) was grown in LB and CVD 103-HgR-SmR was derived from an isolate that exhibited resistance to streptomycin. It was propagated in LB media with streptomycin at 37°C.

#### Preparation of bacteria for inoculation into antibiotic treated mice

Female adult CD-1 mice were given an antibiotic cocktail *ad libidum* (1 g/L ampicillin, 1 g/L neomycin, and 125 mg/L vancomycin) (Ichinohe et al., 2011; Rakoff-Nahoum et al., 2004) for 1 week as described previously with modifications as mice refrained from drinking water with metronidazole (Reikvam et al., 2011). 2.5 g/L of Splenda was added as well to make the cocktail more palatable. 3 days prior to gavage with *V. cholerae*, the cocktail was replaced with 2.5 g/L streptomycin and 2.5 g/L Splenda. Each anaerobic human gut bacterium was cultured from glycerol stocks in LYHBHI media for 24 hours at 37°C, and then diluted (1:50) in fresh LYHBHI media. *Enterococcus faecalis* and *Escherichia coli* were grown aerobically in LYHBHI and LB, respectively, for 24 hours at 37°C, and then diluted (1:50) in respective media. After growth for an additional 48 hours, cultures were normalized for density by OD_600_. For inoculation into adult mice, normalized mixtures were prepared so the equivalent total of 300 μL of OD_600_=0.4 culture divided evenly across the respective strains for each community was pooled, centrifuged, and resuspended in LYHBHI. The suspension was prepared so that each mouse received 50 μL of the bacterial community mixture, as well as 50 μL containing ∼5 x 10^9^ *V. cholerae* O1 El Tor C6706. Prior to bacterial introduction, the mice were fasted for 3 hours and then gavaged with 100 μL of 1 M NaHCO_3_, to buffer stomach acid, after which the bacterial communities and *V. cholerae* were inoculated via oral gavage.

#### DNA extraction

DNA extraction from fecal pellets was done using a combination of mechanical disruption and phenol/chloroform isolation followed by isopropanol precipitation. In brief, fecal pellets were added to sterile 1.8 mL o-ring tubes with 0.1 mm zirconia/silica beads (BioSpec). Then, 500 μL of 200 mM NaCl, 200 mM Tris, and 20 mM EDTA was added along with 210 μL of 20% SDS and 500 μL phenol:chloroform:isoamyl alcohol (25:24:1) (Fisher Biosciences). The microbial cells were lysed via mechanical disruption with a bead beater (BioSpec) for 4 minutes at 2500 RPM. After density separation by centrifugation, the supernatant was again extracted with phenol:chloroform:isoamyl alcohol. The DNA was precipitated with isopropanol with the addition of 3 M sodium acetate at -80C for 1 hour followed by a wash of 100% ethanol and resuspension in nuclease-free water.

#### Quantification of 16S copy number density by qPCR

DNA was extracted from fecal pellets as previously described. The reaction consisted of 2 μL of genomic DNA (20 ng per reaction), 10 μL of SYBR Green Master mix (Biorad), 6 μL of nuclease free water, and 1 μL of 10 uM (forward: 5’-CTCCTACGGGAGGCAGCAG-3’), and 1 μL of 10 uM R primer (reverse: 5’-TTACCGCGG CTGCTGGCAC-3’). Cycle conditions were 95°C for 3 min, followed by 39 cycles (95°C for 10 sec, 55°C for 30 sec, 95°C for 10 sec, 65°C for 5 sec, 95°C for 5 sec). A standard curve was generated as described in Ritalahti and Loffler, et al., 2006 (Ritalahti et al., 2006) using *E. coli* BW30045 as the construct.

#### Human gut microbiome 16S meta-analysis

In order to compare the human gut microbiome in Bangladesh under the dysbiotic pressure of diarrhea, and to compare defined model communities with complete human gut microbiomes, we performed a meta-analysis of existing 16S ribosomal RNA gene sequencing studies. Raw sequencing data of the V4 region of the 16S rRNA gene from published studies were used (for accession numbers, see Table S1). We compared samples taken from different phases of cholera in an adult cohort, examining the earliest sample taken during diarrhea after clinical presentation, the last time points of diarrhea, and a sample taken 3 months into recovery from diarrhea. Fecal samples collected from healthy parents of malnourished Bangladesh children were selected as a healthy adult Bangladesh control (Subramanian et al., 2014). Defined community inputs were calculated on the basis of even distribution of all strains in the specific community (NM: 3000 reads/species; DM: 2000 reads/species). All of the sequencing data were collected together and analyzed using the QIIME 1.9.1 software package (Caporaso et al., 2010).

#### Serum vibriocidal assay

Mouse whole blood was collected via tail vein bleeds using heparinized Caraway collection tubes (Fisher Scientific) or cardiac puncture. Blood was centrifuged at 9,000 x g for 10 minutes, and the serum fraction was isolated and stored at -20°C. The vibriocidal titer measurement was done as previously described with minor modifications (Son and Taylor, 2011). In brief, mouse serum was heat inactivated for 30 minutes at 56°C. The heat-inactivated serum was then serially diluted two-fold with phosphate-buffered saline (PBS). Separately, PBS, guinea pig complement serum (Sigma-Aldrich), and ∼ 5 x 10^8^ CFU *V. cholerae* were combined at a ratio of 7:2:1, respectively. The above mixture was then added to the wells containing serially diluted serum and incubated at 37°C for two hours. The resulting dilutions were then plated onto streptomycin (200 μg/mL) LB plates. The vibriocidal titer is the reciprocal of the highest serum dilution which displayed no *V. cholerae* growth.

#### Fecal pellet collection

Fresh fecal pellets were collected from mice, weighed, and placed in 600 μL of PBS in a 2.0 mL screw cap tube. The pellets were disrupted by agitation without beads in a bead beater (BioSpec) for 30 seconds at 1400 RPM. 10-fold serial dilutions of the resulting fecal slurry were then plated onto LB agar with streptomycin to enumerate *V. cholerae* colonization.

#### Analysis of antibody responses by ELISA

100 μL dense overnight culture of *V. cholerae* grown in LB was plated onto high-binding, clear, flat bottom Costar 96 well plates (Corning, Inc) ELISA plates and allowed to bind overnight. 3% bovine serum albumin (BSA) in PBS was used as a blocking solution. Serum was added at a 1:100 dilution and then diluted two-fold. Alternatively, to measure total antibody levels, serum was added at a 1:100 dilution to plates previously coated with unlabeled goat anti-mouse IgA, IgG, IgM (Southern Biotech) and allowed to bind at 37°C for 3 hours. Next, the plates were washed with PBS with 0.001% Tween-20 and PBS. 100 μL of goat anti-mouse HRP conjugated antibodies of either IgA, IgG_1,2A,2B,3_ or IgM (Southern Biotech) were added to 96 well plates at a dilution of 1:4,000 in 3% BSA and incubated overnight at 4°C. After several washes, the plates were developed with the addition of 5 mg o-phenylenediamine dihydrochloride (Thermo Scientific) and stable peroxide substrate buffer (Thermo Scientific); 1 N HCl was used as a stop solution. The plates were read at 490 nm on a Synergy HTX multi-mode reader (BioTek). Endpoint titer was calculated as the observed signal two standard deviations above background signal.

#### Growth inhibition and passive immune protection assay

From a fresh overnight culture of *V. cholerae,* 1 μL of culture was added to LB with enriched or not enriched antibody and incubated for 6 hours at 37°C for 6 hours. After incubation, samples were plated on streptomycin-LB plates in order to enumerate *V. cholerae* growth. Fecal samples from infected animals bearing the NM and DM communities was collected and processed as described previously. Total IgA/IgM fecal antibody was enriched using Protein L magnetic beads according to the manufacturer’s protocol (Pierce Biotech). 50 ng of enriched antibody was bound to ∼1.25 x 10^6^ CFU *V. cholerae* and allowed to bind at 37°C for 1 hour. 4-day old suckling CD-1 mice were gavaged with 30-gauge plastic tubing with 50 μL of antibody/*V. cholerae* mixture. After 18 hours of infection, the animals were sacrificed, and intestines homogenized for *V. cholerae* CFU enumeration on selective medium.

#### Preparation of heat-killed commensal bacteria

Strains from the NM and DM communities were grown in pure cultures and the bacterial suspension was prepared as previously mentioned. The respective bacterial communities were killed by heating in a heat block for 1 hour at 100°C. Bacterial death was confirmed by plating onto solid media and observing lack of growth.

#### Rescue experiment

Adult CD-1 mice were placed on an antibiotic cocktail of ampicillin (1 g/L), neomycin (1 g/L), and vancomycin (125 mg/L) for 1 week to deplete the native murine microflora as previously described. Prior to introduction of model communities, the mice were switched to streptomycin (2.5 g/L). The mice were pre-colonized with the DM model community 4 days before infection with *V. cholerae*. At time of infection, one group was gavaged with the NM group while the other was gavaged with the DM group. At 2 weeks post infection, the mice were placed back on the ampicillin, neomycin, and vancomycin antibiotic cocktail.

#### *In vivo* depletion of CD4^+^ cells

In order to deplete CD4^+^ cells *in vivo*, 100 μg of GK1.5 antibody (Bio X Cell) was administered intraperitoneally every four days. Depletion of CD4^+^ cells in blood was confirmed using a FACS Canto flow cytometer (BD Biosciences) and FITC rat-anti-mouse CD4 (BD Biosciences). Red blood cell lysis was done using ACK lysis buffer and anti-CD16/32 was used as an Fc block. Analysis was done using Flow Jo (BD Biosciences) and Prism (GraphPad). Mice were treated with ampicillin, neomycin, and vancomycin as previously mentioned. 3 days prior to infection, the mice were placed on streptomycin water alone. The mice were infected with ∼5 x 10^9^ CFU *V. cholerae* and serum vibriospecific ELISAs and vibriocidal assays were performed as previously described.

#### Flow cytometry analysis

Upon animal sacrifice, spleens were mechanically broken down with surgical scissors and ground through a 40 μm strainer with a plastic plunger of a 1 mL syringe into a 50 mL conical tube. The strainer was washed with 5 mLs of FACS Buffer (PBS with 3% w/v bovine serum albumin (BSA)). After centrifugation at 176 x g, cells were resuspended in 2 mLs Pharmlyse Buffer for 2 minutes in a 37 C water bath. After incubation, 40 mLs of FACS buffer were added to the samples. Cell viability was assessed using Trypan Blue. To minimize non-specific Fc receptor binding, rat anti-mouse CD16/32 (BD Pharmingen) was used as an Fc block. Splenic cells were stained with PE rat anti-mouse CD19 (BD Pharmingen), PE-Cy7 anti-mouse B220 (Invitrogen), FITC rat anti-mouse CD3, and APC anti-mouse CD4 (Invitrogen).

### Quantification and statistical analysis

Statistical analyses were performed using GraphPad Prism Software (v9). If data were deemed normally distributed, Student’s *t*-test were performed. If data were deemed not normally distributed, Mann Whitney *U* tests were performed. Statistical details of the experiment can be found in the figures and figure legends.

## Data Availability

• This paper analyses existing, publicly available data. These accession numbers for the datasets are listed in the key resources table. • This paper does not report original code • Any additional information required to reanalyze the data reported in this paper is available from the lead contact upon request.
