# Supplementary material for: A dysbiotic gut microbiome suppresses antibody mediated-protection against Vibrio cholerae
Source: iScience. 2021 Nov 14;24(12):103443. doi: 10.1016/j.isci.2021.103443 (PMC8633975; doi:10.1016/j.isci.2021.103443)
Supplement: Document S1. Figures S1 and S2 and Table S1 [file mmc1.pdf]

iScience, Volume *24*

Supplemental information

A dysbiotic gut microbiome suppresses antibody mediated-protection against *Vibrio cholerae*

John C. Macbeth, Rui Liu, Salma Alavi, and Ansel Hsiao

Figure S1

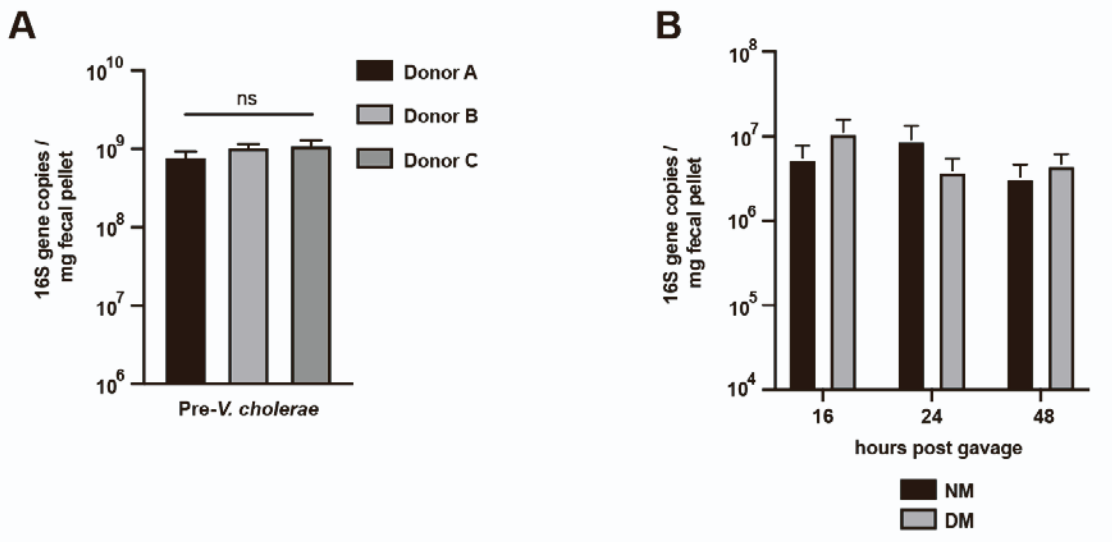

Figure S1. 16S gene copy number per mg fecal pellet for designated samples, Related to Figures 1 & 2. (A) After 2 weeks of colonization in germ-free mice, little variation in bacterial abundance was observed by 16S qPCR prior to infection with *V. cholerae*. (B) Bacterial abundance in the antibiotic treated adult mouse model post gavage with indicated communities. ns, $P>0.05$, one-way ANOVA.

Figure S2

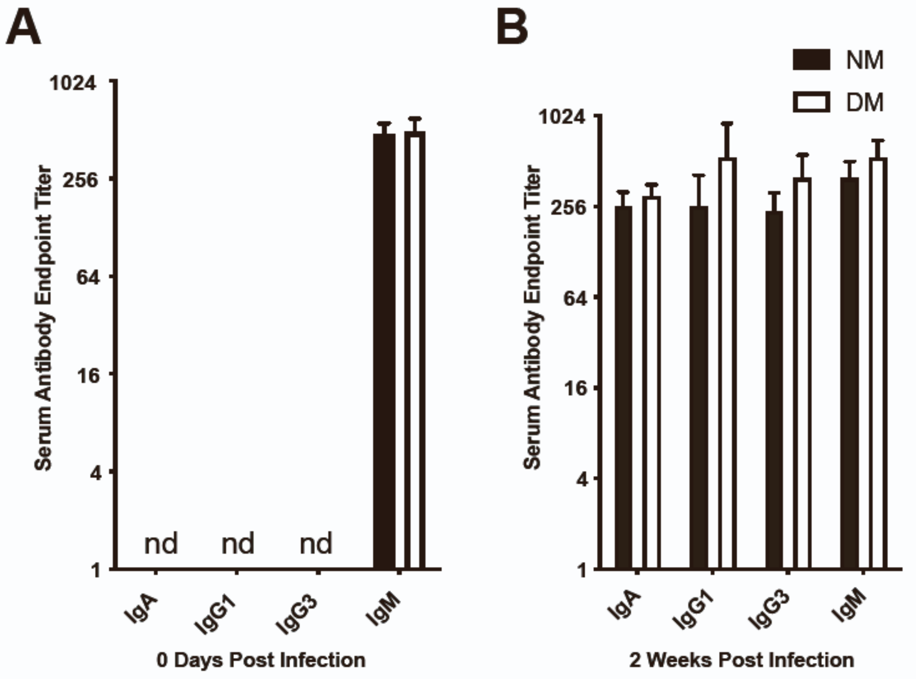

Figure S2. Serum antibody profiles at designated timepoints, Related to Figure 2. (A) Serum antibody profiles against whole cell *V. cholerae* 0 days post infection and (B) 2-weeks post-infection. nd, not detected.

Table S1. Public datasets used for 16S rRNA meta-analysis, Related to STAR Methods

Run Accession	Fecal Sample ID	ENA Accession Number	Group
ERR520665	A.diarrhea.016	PRJEB6358	Diarrhea (start)
ERR520694	A.diarrhea.end	PRJEB6358	Diarrhea (end)
ERR520710	A.recovery.d088	PRJEB6358	Recovery (end)
ERR520711	B.diarrhea.018	PRJEB6358	Diarrhea (start)
ERR520728	B.diarrhea.end	PRJEB6358	Diarrhea (end)
ERR520744	B.recovery.d088	PRJEB6358	Recovery (end)
ERR520745	C.diarrhea.011	PRJEB6358	Diarrhea (start)
ERR520825	F.diarrhea.005	PRJEB6358	Diarrhea (start)
ERR520851	F.diarrhea.end	PRJEB6358	Diarrhea (end)
ERR520867	F.recovery.d087	PRJEB6358	Recovery (end)
ERR520868	G.diarrhea.005	PRJEB6358	Diarrhea (start)
ERR520884	G.diarrhea.028	PRJEB6358	Diarrhea (end)
ERR498974	Bgtw10.F.m13	PRJEB5482	Healthy Bangladesh
ERR499132	Bgtw10.M.m13	PRJEB5482	Healthy Bangladesh
ERR499151	Bgtw11.M.m12	PRJEB5482	Healthy Bangladesh
ERR498802	Bgtw12.F.m4	PRJEB5482	Healthy Bangladesh
ERR499027	Bgtw2.M.m24	PRJEB5482	Healthy Bangladesh
ERR499445	Bgtw3.F.m16	PRJEB5482	Healthy Bangladesh
ERR499505	Bgtw3.M.m22	PRJEB5482	Healthy Bangladesh
ERR498846	Bgtw4.F.m13	PRJEB5482	Healthy Bangladesh
ERR499050	Bgtw4.F.m13	PRJEB5482	Healthy Bangladesh
ERR498850	Bgtw4.M.m20	PRJEB5482	Healthy Bangladesh
ERR499054	Bgtw4.M.m20	PRJEB5482	Healthy Bangladesh
ERR498702	Bgtw5.F.m13	PRJEB5482	Healthy Bangladesh
ERR499227	Bgtw5.M.m22	PRJEB5482	Healthy Bangladesh
ERR498551	Bgtw6.F.m10	PRJEB5482	Healthy Bangladesh
ERR499464	Bgtw6.M.m10	PRJEB5482	Healthy Bangladesh
ERR499518	Bgtw6.M.m10	PRJEB5482	Healthy Bangladesh
ERR498561	Bgtw7.F.m7	PRJEB5482	Healthy Bangladesh
ERR498884	Bgtw7.M.m16	PRJEB5482	Healthy Bangladesh
ERR499088	Bgtw7.M.m16	PRJEB5482	Healthy Bangladesh
ERR499476	Bgtw8.F.m13	PRJEB5482	Healthy Bangladesh
ERR499530	Bgtw8.F.m13	PRJEB5482	Healthy Bangladesh
ERR499249	Bgtw8.M.m13	PRJEB5482	Healthy Bangladesh
ERR498905	Bgtw9.F.m13	PRJEB5482	Healthy Bangladesh
ERR499109	Bgtw9.F.m13	PRJEB5482	Healthy Bangladesh
ERR498908	Bgtw9.M.m13	PRJEB5482	Healthy Bangladesh

ERR499112	Bgtw9.M.m13	PRJEB5482	Healthy Bangladesh
ERR3190459	SA.Human1.Sample	PRJEB31497	Donor A
ERR3190460	SA.Human2.Sample	PRJEB31497	Donor B
ERR3190463	SA.Human6.Sample	PRJEB31497	Donor C
